# Gold Nanocomplex Strongly Modulates the PI3K/Akt Pathway and Other Pathways in MCF-7 Breast Cancer Cell Line

**DOI:** 10.3390/ijms21093320

**Published:** 2020-05-08

**Authors:** Nouf N. Mahmoud, Duaa Abuarqoub, Rand Zaza, Dima A. Sabbah, Enam A. Khalil, Rana Abu-Dahab

**Affiliations:** 1 Department of Pharmacy, Faculty of Pharmacy, Al-Zaytoonah University of Jordan, Amman 11733, Jordan; dima.sabbah@zuj.edu.jo; 2 Cell Therapy Center, The University of Jordan, Amman 11942, Jordan; randzaza@gmail.com; 3 Department of Pharmacology and Biomedical Sciences, Faculty of Pharmacy and Medical Sciences, University of Petra, Amman 11196, Jordan; 4 School of Pharmacy, The University of Jordan, Amman 11942, Jordan; ekayoub@ju.edu.jo (E.A.K.); abudahab@ju.edu.jo (R.A.-D.)

**Keywords:** gold nanorods, PI3K inhibitor, nanocomplex, transcription factors, regulatory proteins, PI3K/Akt pathway

## Abstract

Conjugating drugs with gold nanoparticles (GNP) is a key strategy in cancer therapy. Herein, the potential inhibition of the phosphatidylinositol 3-kinase (PI3K)/Akt pathway, and other pathways of the MCF-7 cell-line, was investigated upon treatment with gold nanorods (GNR) conjugated with a PI3K inhibitor drug. The results revealed that the coupling of GNR with the drug drastically modulated the expression of PI3Kα at the gene and protein levels compared to the drug or GNR alone. The PI3Kα pathway is involved in tumor progression and development through the mediation of different mechanisms such as apoptosis, proliferation, and DNA damage. Treatment with the nanocomplex significantly affected the gene expression of several transcription factors responsible for cell growth and proliferation, apoptotic pathways, and cell cycle arrest. Furthermore, the gene expression of different regulatory proteins involved in cancer progression and immune responses were significantly modified upon treatment with the nanocomplex compared to the free drug or GNR alone.

## 1. Introduction

Gold-based nano-formulations have significant applications in oncology, including diagnosis, imaging, and treatment of cancers, due to their outstanding physical and optical merits [1,2,3]. Gold nanoparticles (GNP) could be tuned into several shapes and sizes, and their surfaces could easily be coupled with several small ligands and biomolecules that greatly affect their interaction and uptake into cancer cells [4,5,6]. Conjugating GNP with anti-cancer agents is considered to be a cornerstone in nanotechnology-based cancer therapy. Such drug conjugation improves the pharmacokinetics and anti-cancer efficiency of the payload, and consequently minimizes the adverse reactions [7,8]. The coupling of drugs with GNP of different shapes, sizes and surface chemistries for anticancer activity was extensively reported in the literature [5,9,10,11,12,13]. However, the exact mechanisms of action of the conjugates at the molecular level are not fully discussed in the majority of the published studies. A recent study indicates that GNP synthesized by green chemistry activates the caspase expression and downregulates the anti-apoptotic protein expression in the A549 lung cancer cell line [14]. Furthermore, GNP conjugated with resveratrol induced cell cycle arrest in the MCF-7 cell line, as described by Lee et al. [15]. In our recent study, the anti-cancer activity of a new novel compound having phosphatidylinositol 3-kinase (PI3Kα) inhibitory activity; N-(4-hydroxyphenyl)4-hydroxy-2-quinolone-3-carboxamide [16], was drastically enhanced against the MCF-7 breast cancer cell line upon conjugation with cholesterol-coated gold nanorods (GNR), and the obtained nanocomplex showed rapid effects on the cell death cycle [17]. PI3Kα induces cell growth and division by enhancing the synthesis of triphosphates [18]. Herein, we explain the potent anti-cancer activity of the nanocomplex against the MCF-7 breast cancer cell line by providing insights into the mechanisms of cytotoxicity at the molecular level. The inhibition potential of the nanocomplex on the PI3Kα/Akt pathway and other related pathways was investigated and compared to that of GNR, or the drug alone at the gene level, using polymerase chain reaction (PCR) array and at the protein level using confocal microscopy and flow cytometry.

## 2. Results and Discussion

In our recent work, coupling of the PI3kα inhibitor with cholesterol-coated GNR resulted in substantial potent anti-proliferative activity of the nanocomplex against the MCF-7 cell line, compared to the effect of the GNR or the drug alone. The PI3kα inhibitor compound used in this study was N-(4- hydroxyphenyl)4-hydroxy-2-quinolone-3-carboxamide, one of a new series of N-substituted-4- hydroxy-2-quinolone-3-carboxamides compounds, that were synthesized, characterized, and biologically tested previously in our lab, and displayed promising phosphatidylinositol 3-kinase (PI3Ka) inhibitory activity [16]. The conjugated GNR-PI3kα inhibitor nanocomplex demonstrated a direct impact on cell death modality, and it was a strong inhibitor for cell cycle progression [17]. In the current study, we are explaining the mechanism of cytotoxicity of the nanocomplex and its inhibition potential on the PI3Kα/Akt pathway and other related pathways, compared to that of GNR or the drug alone at the gene and protein levels.

At the gene level, Ingenuity Pathway Analysis (IPA) software (Qiagen, USA) was used to measure the changes in the expression levels of a number of genes that are involved in the PI3Kα/Akt pathway, and to determine the subsequent predicted outcomes. The investigated 84 genes play major roles in the PI3K/Akt pathway, and alteration in the expression level of any of these genes apart from the normal expression level would impact the overall outcome of the pathway.

**Figure 1:**
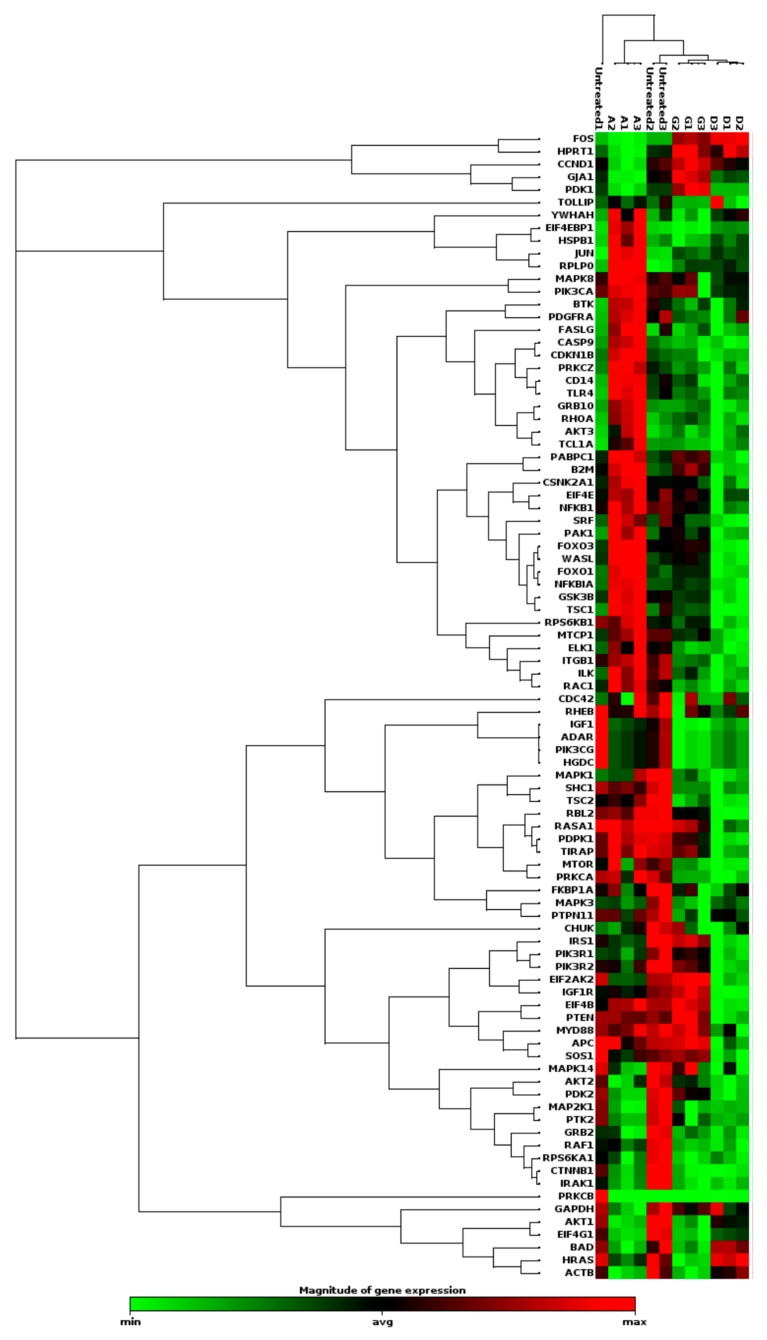
PCR array analysis of PI3K/Akt signaling pathway of MCF-7 cells. A representative heat map of expression values of genes involved in PI3K pathways among different treated groups; the nanocomplex, the free drug and gold nanorods (GNR) only (presented in the map as A: nanocomplex; D: the free drug and G: GNR) compared to their control untreated cells.

Figure 1 shows a heat map representing the variation in the expression values of all genes that are part of PI3K pathway upon treatment with the nanocomplex, GNR or the free drug.

IPA analysis demonstrated that the nanocomplex has shown a high impact on the downstream genes of the PI3K/Akt pathway targeting the catalytic subunits (Figure S1, Supplementary Materials), while the single treatment (free drug or GNR) has an effect on the upstream genes of the PI3K/Akt pathway (Figures S2 and S3, Supplementary Materials), which denotes different targets for the compared treatments. The quantitative analysis of fold changes in the expression of the derived genes among all groups, showed that the nanocomplex has the highest impact on the expression levels of these genes compared to the single treatment of the free drug or GNR, as described in Table S1, Supplementary Materials.

Regarding these results, the nanocomposite is shown to have direct effects on various biological processes, such as the cell progression and entry into the cell cycle, cytoskeleton re-organization, differentiation, and metabolism. The targeted genes involved in the PI3K/Akt pathway that were significantly affected by the treatment are classified into categories as the following:

### 2.1. Transcription Factors

The IPA upstream regulator analytics suggests a predicted activation of the transcription factor Forkhead Box O (FoxO1), and to a lesser extent FoxO3, upon treatment with the nanocomplex (Figure S1, Table S1, Supplementary Materials). On the other hand, the free drug treatment showed a predicted inhibition of the transcription factors FoxO1 and FoxO3, whereas treatment with GNR did not affect the expression of these transcription factors (Figures S2 and S3, Table S1, Supplementary Materials). The FoxO transcription factors are considered as downstream targets of the Akt signaling pathway, which are involved in growth inhibition, apoptosis, and the passage of cells through the cell cycle, in addition to their role in some tissue-specific metabolic dysregulations [19,20]. Additionally, our results showed that treatment with the nanocomplex suppressed the expression of the TSC2 complex, which is a potent inhibitor of mTORC1 that promotes cell growth and proliferation; thus, it functions mainly against Akt activity, as its downregulation has a negative feedback effect (Figure S1, Table S1, Supplementary Materials). The free drug and GNR treatments showed the same effect on the TSC complex (Figures S2 and S3, Table S1, Supplementary Materials).

### 2.2. Regulatory Proteins

The effect of the nanocomplex in upregulating the IkB/NF-KB complex, which is localized in the cytoplasm, is interesting as this will affect the NF-kB-mediated transcription with suggested consequences on cell proliferation, survival, and immune responses, since NF-kB is considered to be the main coordinator of cell growth, adhesion, apoptosis, and immune and inflammatory responses [21]. On the other hand, the free drug and GNR treatments showed an opposite effect on the expression of these genes. Our analysis showed that the downregulation was predicted for the IkB/NF-KB complex localized in the nucleus, while in the case of nanocomplex treatment, the complex was localized in the cytoplasm, and this could explain the variation in the expression pattern of these genes (Figures S1–S3, Table S1, Supplementary Materials). Furthermore, The PTEN signaling pathway, which has an opposing effect to the Akt, has many genes that are affected by treatment with the nanocomplex, such as the downregulation of FAK, Akt, and Cyclin D1, and the upregulation of FoxO and FASL, which result in increased cell apoptosis [22] (Figure S1, Table S1, Supplementary Materials). The data from the PCR array displays upregulation of FASLG upon treatment with the nanocomplex, which reflects on the downstream consequence of DNA damage that enhances cell death (Table S1, Supplementary Materials).

Moreover, the inhibition of PI3k and its catalytic subunits upon treatment with the nanocomplex through the downregulation of the product genes PIK3CA, PIK3CB, PIK3CG, and PIK3CD, results in the dysregulation of the regulatory function of HRAS, which is responsible for regulating many cellular functions such as cell proliferation, differentiation, apoptosis, and senescence [23]. On the other hand, downregulation of AKT, JNK, and CASP9 in the Myc-mediated apoptosis signaling reflects the final apoptotic outcome for cancer cells [24]. The 84 gene expression profiles upon treatment with the nanocomplex could be explained by a predicted inhibition of HRAS that leads to the inhibition of the PI3K complex with downstream inhibition of ESR1, STAT1, and NFkB1. A predicted inhibition of EGFR leads to changes in the expression levels of JUN and HIF1A, resulting in the dysregulation of different cellular processes, such as cell growth, cell cycle entry, cell survival, cytoskeleton reorganization, and metabolism [23].

Thus, we can conclude that the crosstalk between the PI3K/Akt pathway and other pathways is very essential to regulate the components and downstream targets that are involved in other pathways. Our findings showed that the nanocomplex displays a significant inhibition of P13K and other related pathways that play a role in tumor progression and development through the mediation of different mechanisms such as apoptosis, proliferation, and DNA damage. This high detection impact on these pathways was only achieved upon treatment with the nanocomplex, not with the free drug or GNR.

To confirm that the inhibitory effect of these treatments (the nanocomplex, the free drug, and GNR) is not only restricted to the gene level, the expression of PI3Kα at the protein level was explored after treating cells with the aforementioned treatments, by using confocal microscopy and flow cytometry.

**Figure 2:**
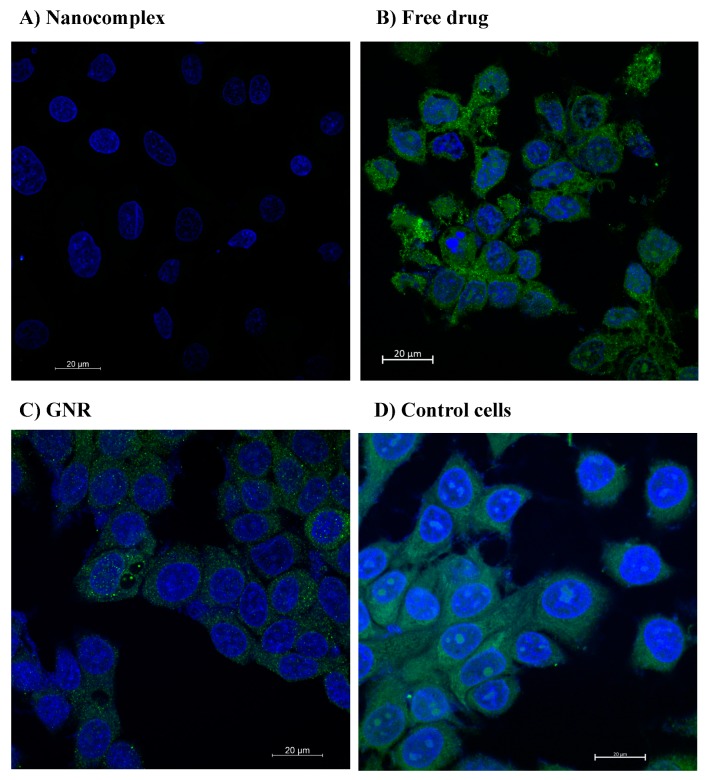
The inhibitory effect of the nanocomplex (**A**), free drug (**B**) and GNR (**C**) on the expression of PI3Kα protein activity of MCF-7 breast cancer cells compared to control untreated cells (**D**). Treatment with the nanocomplex drastically inhibits the expression of PI3Kα compared to the free drug or GNR. Blue color indicates nuclei of the cells were stained with DAPI, and green color indicates PI3Kα antibody was stained with Alexaflour488.

The results demonstrated that the nanocomplex was able to show a complete inhibition of the PI3Kα expression pattern compared to other treatments. However, treatment with the free drug showed a slight decrease in the expression signal of PI3Kα compared to the control cells, while GNR treatment showed similar expression pattern of PI3Kα to the control untreated cells (Figure 2).

**Figure 3:**
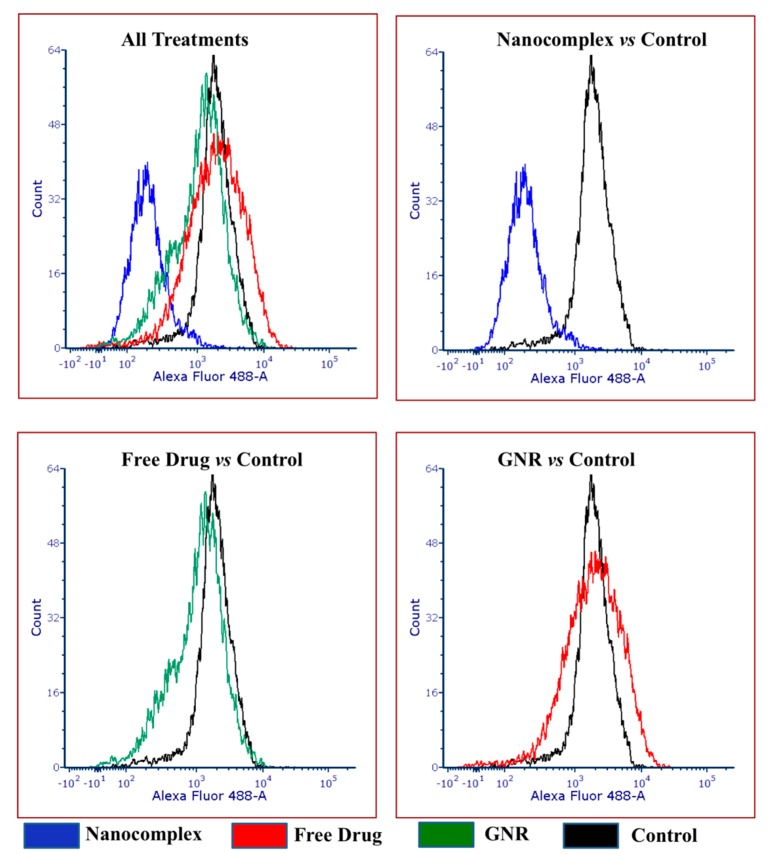
Flow cytometric histograms, representing the expression of PI3Kα of MCF-7 cells treated with nanocomplex, free drug and GNR. Treatment with the nanocomplex significantly inhibits the expression of PI3Kα of MCF-7 cancer cell line compared to other treatments.

**Figure 4:**
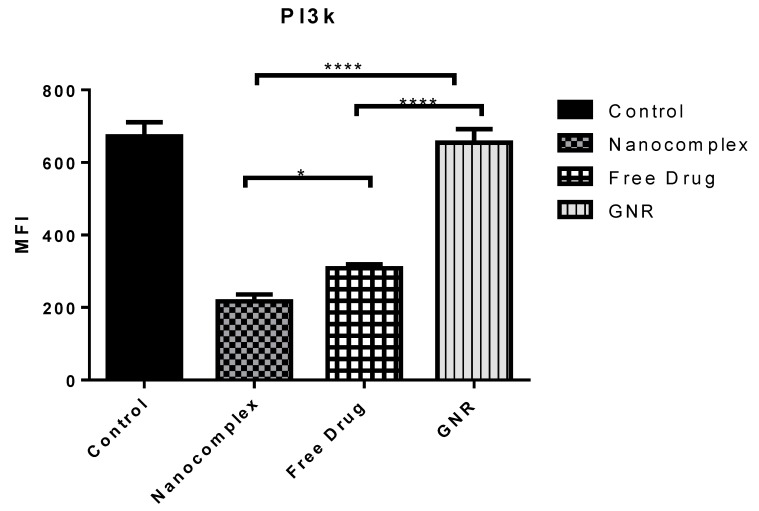
Statistical analysis of flow cytometric results, measuring the mean fluorescence intensity of PI3Kα expression (MFI) among all treated groups compared to their control untreated cells. Data are represented as mean ± SD, n = 3. A one-way analysis of variance (ANOVA) test was performed followed by Tukey’s multiple comparisons test; * *p* < 0.05, **** *p* < 0.0001.

Interestingly, the data from the flow cytometry are consistent with the previously mentioned data from confocal microscopy. The nanocomplex showed a great inhibitory effect on PI3Kα protein activity compared to the other single treatments, as it demonstrated a significant decrease in the expression level of PI3Kα by 3-fold compared the control untreated group (*p* < 0.05) (Figure 3 and Figure 4). In contrast, the free drug treatment decreased the expression activity by 2-fold compared to the control untreated group (*p* < 0.05). Alternatively, cells treated with GNR showed similar expression activity of PI3Kα to the untreated control cells (Figure 3 and Figure 4).

Thus, we can conclude that the GNR significantly enhanced the activity of the conjugated drug, as the inhibitory effect of the nanocomplex on the PI3Kα/Akt and other related pathways was drastically enhanced compared to the free drug or GNR treatment.

The current study provides molecular evidence that explains the potent effect of the nanocomplex on the proliferation and apoptosis of cancer cells. Treatment with the nanocomplex demonstrated a dramatic inhibition of the P13K/Akt and other related pathways involved in tumor progression and development through the mediation of different mechanisms such as apoptosis, proliferation, and DNA damage at the gene level. Additionally, treatment with the nanocomplex resulted in complete inhibition of the target PI3K at the protein level.

The high detection impact on these pathways, indicating the high potential of our combined therapy to target the oncogenesis pathway, makes this nanocomplex considerable as a very promising platform for targeted breast cancer therapy.

## 3. Material and Methods

### 3.1. Synthesis and Characterization of Cholesterol-GNR Coupled with PI3K∝ Inhibitor

GNR were synthesized using the seed-mediated method, functionalized with PEGylated-thiolated cholesterol moiety, and then conjugated with the PI3K∝ inhibitor drug N-(4- hydroxyphenyl)4-hydroxy-2-quinolone-3-carboxamide and fully characterized as described previously [17].

### 3.2. Polymerase Chain Reaction (PCR) Array

RNA was extracted from pre-treated MCF-7 cells using the Trizol-hybrid method (Qiagen, Germantown, MD, USA); the nanocomposite (GNR; 0.06 nM, drug; 6 µg/mL), the drug (6 µg/mL), and GNR (0.06 nM) were applied for 24 h prior to extraction. A total of 0.5 μg extracted RNA was converted to cDNA by using RT^2^ First Strand Kit (Qiagen, Germantown, MD, USA). Then, cDNA samples were diluted and amplified with the RT^2^ SYBR^®®^ green master mix of (PAHS-058Z, RT² Profiler™ PCR Array Human PI3K/Akt pathway, Qiagen, Germantown, MD, USA) according to the manufacturer’s instructions, and loaded into the 96-well array. The amplification conditions were as follows; 95 °C for 10 min, then 40 cycles of 95 °C for 15 sec, and 60 °C for one min. Samples were run on a CFX 96 C1000 system (Biorad, Hercules, CA, USA). Data were analyzed automatically according to the SABiosciences company (Qiagen, Germantown, MD, USA) web portal www.SABiosciences.com/pcrarraydataanalysis.php by using the ΔΔCt method. The expression levels of the genes were normalized to the following housekeeping genes: Beta-2-microglobulin (B2M), hypoxanthine phosphoribosyl transferase 1 (HPRT1), and actin beta (ACTB). For data analysis, the differential expression level of the apoptotic genes was identified using Student’s t-test (two-tailed, unpaired). A cut-off point of 1.5 was used as a threshold to determine the statistical significance of the upregulated or downregulated genes (*p* < 0.05). The analysis of the PCR array data was performed using the Ingenuity Pathway Analysis software (Qiagen, Germantown, MD, USA).

### 3.3. Confocal Microscopy

Confocal microscopy was used to explore the impact of the following treatments—nanocomplex, free drug, and GNR—on the protein activity of PI3kα.

MCF-7 cells (4 × 10^5^ cells) were seeded into coverslips until reaching confluency. Then, cells were treated with the following treatments: the nanocomplex, GNR, and the drug alone. Cells cultured in the growth media without the addition of any treatment were used as control untreated cells.

After 24 h of treatment, the cells were fixed by 4% paraformaldehyde (PFA) for 20 min at room temperature (RT). Then, the PFA was removed and cells were washed with the washing buffer (0.1% triton X diluted in phosphate-buffered saline (PBS)). Then, cells were permeabilized with 0.3% triton X and incubated for 15 min at RT. Next, the permeabilization solution was removed and the blocking solution was added followed by incubation for 30 min at RT. After that, the blocking solution was removed and the PI3Kα primary antibody (Abcam, Eugene, OR, USA) diluted in blocking solution was added according to the manufacturer’s recommended concentration and incubated overnight at 4 °C. On the next day, the solution was removed, and coverslips were washed thrice with the washing buffer (0.1% Triton X). Following that, secondary antibodies diluted in the blocking solution were added and incubated for 1 h at 37 °C. Then, the solution was removed, and coverslips were washed thrice with the washing buffer. 4′,6-diamidino-2-phenylindole (DAPI) stain (Thermofisher, Waltham, MA, USA) was added to the cells and incubated for 5 min, followed by a washing step. Finally, coverslips were transferred into glass slides loaded with one drop of mounting media (DAKO, Glostrup, Denmark). At last, confocal images were acquired via a laser scanning microscope (LSM) 780 (Zeiss, Oberkochen, Germany). The objective used for acquiring the images was a Plan-Apochromat 63X/1.4 Oil DIC M27. Lasers of 405 nm and 488 nm were activated for excitation of the nuclear stain DAPI, and Alexaflour488 when present, respectively. Detector ranges for emission signals were: 410–556 nm for DAPI, and 525 nm for Alexaflour488.

### 3.4. Flow Cytometry

MCF-7 cells (American Type Culture Collection (ATCC), USA) (4 × 10^5^ cells) were seeded into 6-well plates (Greiner Bio-One, Frickenhausen, Germany) until reaching confluency. Then, cells were treated with the following: the nanocomplex, GNR, and the drug. Cells cultured in the growth media without the addition of any treatment were used as control untreated cells.

After 24 h, cells were collected by trypsin EDTA 0.25% (Euroclone, Milan, Italy), and washed with phosphate-buffered saline (PBS). Following that, cells were centrifuged at 400× *g* for 5 min. Supernatant was removed and cells were resuspended in 1 mL paraformaldehyde (PFA) (2%) and incubated for 10 min at room temperature (RT). For permeabilization, cells were centrifuged and resuspended in 1 mL 100% ice cold methanol at −20 °C for 30 min. Next, cells were centrifuged and resuspended with Flow Cytometry Lysing Solution (FACS) stain buffer (BD Biosciences, NJ, USA). Then, cells were treated with the diluted PI3Kα primary antibody 1:100 (Abcam, USA) in the dark at RT on a plate shaker for 40 min. Afterwards, 1.0 mL of cell wash solution (BD, USA) was added to the stained cells and centrifuged at 400× *g* for 5 min. Following that, cells were suspended with 50 µL of FACS stain buffer and stained with the secondary antibody IgG Alexaflour 488 (Abcam, Eugene, OR, USA) in the dark at RT on a plate shaker for 40 min. Incubated cells were washed twice with cell wash solution and then centrifuged at 400× *g* for 5 min. At the end, cells were resuspended with 200 uL of PBS. The expression profile was analyzed by the Fluorescein Activated Cell Sorter FACS Canto II (BD Biosciences, NJ, USA). Mean fluorescence intensity (MFI) was used to measure the differences in the expression profile of PI3Kα.

### 3.5. Statistical Analysis

Statistical analysis was performed using the one-way analysis of variance (ANOVA) test followed by Tukey’s multiple comparisons using GraphPad Prism, version 8.1.

## Supplementary Materials

The following are available online at https://www.mdpi.com/1422-0067/21/9/3320/s1. PI3k-Akt pathway of MCF-7 cells treated with the nanocomplex (Figure S1), the free drug (Figure S2) and the GNR (Figure S3). Table S1: Summary of PCR array results of fold changes and the statistical significance of PI3K/Akt pathway genes, in MCF-7 cells treated with; nanocomplex, free drug only and GNR only, compared to their control untreated cells.
