# Supplementary material for: Gold Nanocomplex Strongly Modulates the PI3K/Akt Pathway and Other Pathways in MCF-7 Breast Cancer Cell Line
Source: Int J Mol Sci. 2020 May 8;21(9):3320. doi: 10.3390/ijms21093320 (PMC7246767; doi:10.3390/ijms21093320)
Supplement: Supplementary file 1 [file ijms-21-03320-s001.pdf]

Supplementary Materials

Gold Nanocomplex Strongly Modulates the PI3K/Akt Pathway and Other Pathways in MCF-7 Breast Cancer Cell Line

Nouf N. Mahmoud [1,*,†], Duaa Abuarqoub [2,3,*,†], Rand Zaza [2], Dima A. Sabbah [1], Enam A. Khalil [4] and Rana Abu-Dahab [4]

[1] Department of Pharmacy, Faculty of Pharmacy, Al-Zaytoonah University of Jordan, Amman 11733, Jordan; dima.sabbah@zuj.edu.jo
[2] Cell Therapy Center, The University of Jordan, Amman 11942, Jordan; randzaza@gmail.com
[3] Department of Pharmacology and Biomedical Sciences, Faculty of Pharmacy and Medical Sciences, University of Petra, Amman 11196, Jordan
[4] School of Pharmacy, The University of Jordan, Amman 11942, Jordan; ekayoub@ju.edu.jo (E.A.K.); abudahab@ju.edu.jo (R.A.-D.)
* Correspondence: nouf.mahmoud@zuj.edu.jo (N.N.M.); duaa.abuarqoub@uop.edu.jo (D.A.)
† These authors contributed equally to this work.

Table S1: Summary of PCR array results of fold changes and the statistical significance of PI3K/Akt pathway genes, in MCF-7 cells treated with; nanocomplex, free drug only and GNR only, compared to their control untreated cells.

		Nanocomplex		Free drug		GNR	
**Name of the gene**	**Ref Seq**	**Fold change**	**P-value**	**Fold change**	**P-value**	**Fold change**	**P-value**
**ADAR**	NM_001111	-1.3051	0.03881	-1.5228	0.01126	-1.8	0.00445
**BTK**	NM_000061	2.251	0.02933	-1.4781	0.5028	-1.1893	0.53463
**CD14**	NM_000591	2.2275	0.00842	-1.6437	0.23034	-1.2436	0.57322
**EIF2AK2**	NM_002759	-1.4308	2.1E-05	-1.8529	9.2E-05	1.0872	0.01077
**FASLG**	NM_000639	2.6854	0.02258	-1.4324	0.39797	-1.0765	0.74283
**FOXO1**	NM_002015	2.1567	0.00024	-1.8855	0.00475	1.2211	0.03383
**FOXO3**	NM_001455	1.6199	0.00023	-2.052	0.00044	1.1176	0.07485
**CCND1**	NM_053056	-1.7735	-1.0123	1.2568	0.00037	0.856224	0.01034
**FOS**	NM_005252	-1.4009	0.00055	2.826	1.8E-05	2.4197	1.7E-05
**MTCP1**	NM_001018025	1.1943	0.13125	-1.6117	0.00817	-1.1029	0.27334
**MYD88**	NM_002468	-1.0621	0.3897	-1.5111	0.02002	-1.0167	0.7535
**PIK3CG**	NM_002649	-1.3051	0.03881	-1.5228	0.01126	-1.8	0.00445
**TIRAP**	NM_001039661	1.0719	0.49421	-2.5611	0.00134	-1.1973	0.17231
**TOLLIP**	NM_019009	1.0257	0.82705	-1.0721	0.96737	-1.2446	0.06496
**HSPB1**	NM_001540	1.8283	0.00305	1.1906	0.05121	-1.0899	0.32681
**PTEN**	NM_000314	-1.0142	0.69054	-1.7438	0.00037	1.1014	0.17667
**PTK2**	NM_005607	-1.6173	0.0016	-1.6245	0.00109	-1.4912	0.02711
**SHC1**	NM_003029	-1.1218	0.02928	-1.5407	0.00125	-1.4417	0.00046
**CASP9**	NM_001229	1.979	0.00069	-1.3475	0.02069	-1.2161	0.08741
**PDGFRA**	NM_006206	1.9789	0.1086	-1.5618	0.59832	-1.3068	0.38237
**PDPK1**	NM_002613	1.0539	0.42872	-1.9931	0.0005	-1.2091	0.04431
**IGF1**	NM_000618	-1.3051	0.03881	-1.5228	0.01126	-1.7618	0.00464
**IGF1R**	NM_000875	-1.1556	0.18786	-2.448	0.00196	1.243	0.07504
**AKT1**	NM_005163	-1.6362	0.00053	-1.2491	0.00824	-1.6029	0.00131
**AKT2**	NM_001626	-1.447	0.01346	-1.5465	0.00189	-1.2779	0.01493
**AKT3**	NM_005465	4.3447	0.01683	1.0699	0.64665	1.3247	0.3346
**APC**	NM_000038	-1.1353	0.30256	-2.0737	0.00277	1.0169	0.74446
**CDKN1B**	NM_004064	1.5608	0.0003	-1.1962	0.00824	-1.1517	0.15632
**CHUK**	NM_001278	-1.5319	0.26589	-2.3415	0.14592	-1.9199	0.33174
**EIF4E**	NM_001417	1.3405	0.05769	-1.6197	0.09247	-1.0155	0.84644
**EIF4EBP1**	NM_001968	2.8238	0.00032	1.3306	0.00035	-1.1406	0.01744
**EIF4G1**	NM_004095	-2.1396	0.00262	-1.5161	0.01305	-2.1664	0.003
**GJA1**	NM_000165	-3.1364	0.00029	-1.3238	0.02305	1.5581	0.00638
**IRAK1**	NM_001569	-1.616	0.02428	-1.7167	0.01656	-1.7704	0.01575
**IRS1**	NM_005544	-1.5275	0.02928	-2.3691	0.0054	-1.0124	0.8453
**JUN**	NM_002228	4.8971	2.2E-05	2.3563	0.00051	2.0806	0.00322
**MAPK8**	NM_002750	1.5396	0.00066	-1.3686	0.01482	-2.4578	0.33377

**NFKB1**	NM_003998	1.2191	0.02727	-1.6034	0.00639	-1.1114	0.0855
**NFKBIA**	NM_020529	1.7638	8.8E-05	-1.4718	0.00098	1.1013	0.02352
**PDGFRA**	NM_006206	1.9789	0.1086	-1.5618	0.59832	-1.3068	0.38237
**PDK1**	NM_002610	-1.8341	0.00025	-1.5071	0	1.6977	0.00232
**PDK2**	NM_002611	-2.0253	0.00075	-2.1739	0.00051	-1.2616	0.02208
**PDPK1**	NM_002613	1.0539	0.42872	-1.9931	0.0005	-1.2091	0.04431
**PIK3CA**	NM_006218	1.4399	0.00214	-1.5548	0.00065	-6.7853	0.59124
**PIK3CG**	NM_002649	-1.3051	0.03881	-1.5228	0.01126	-1.8	0.00445
**PRKCA**	NM_002737	-1.0251	0.90408	-1.742	0.00051	-1.5472	0.00052
**PRKCB**	NM_002738	-9.6753	0.37408	-37.8708	0.37384	-21.9354	0.37389
**PTK2**	NM_005607	-1.6173	0.0016	-1.6245	0.00109	-1.4912	0.02711
**RHOA**	NM_001664	1.5597	0.00132	-1.0581	0.32863	1.1294	0.03206
**RPS6KA1**	NM_002953	-1.4863	0.03546	-1.5671	0.01755	-1.6669	0.01021
**TCL1A**	NM_021966	3.8302	0.01496	-1.2102	0.86521	1.0091	0.88079
**TIRAP**	NM_001039661	1.0719	0.49421	-2.5611	0.00134	-1.1973	0.17231
**TLR4**	NM_138554	3.0538	0.00665	-2.0865	0.31813	-1.0924	0.59668
**TSC1**	NM_000368	1.6086	0.00935	-1.4861	0.04942	1.0005	0.91985
**TSC2**	NM_000548	-1.1601	0.26256	-2.1681	0.00648	-1.7772	0.01956
**WASL**	NM_003941	1.6329	5.5E-05	-1.6275	0.00069	1.1015	0.10251

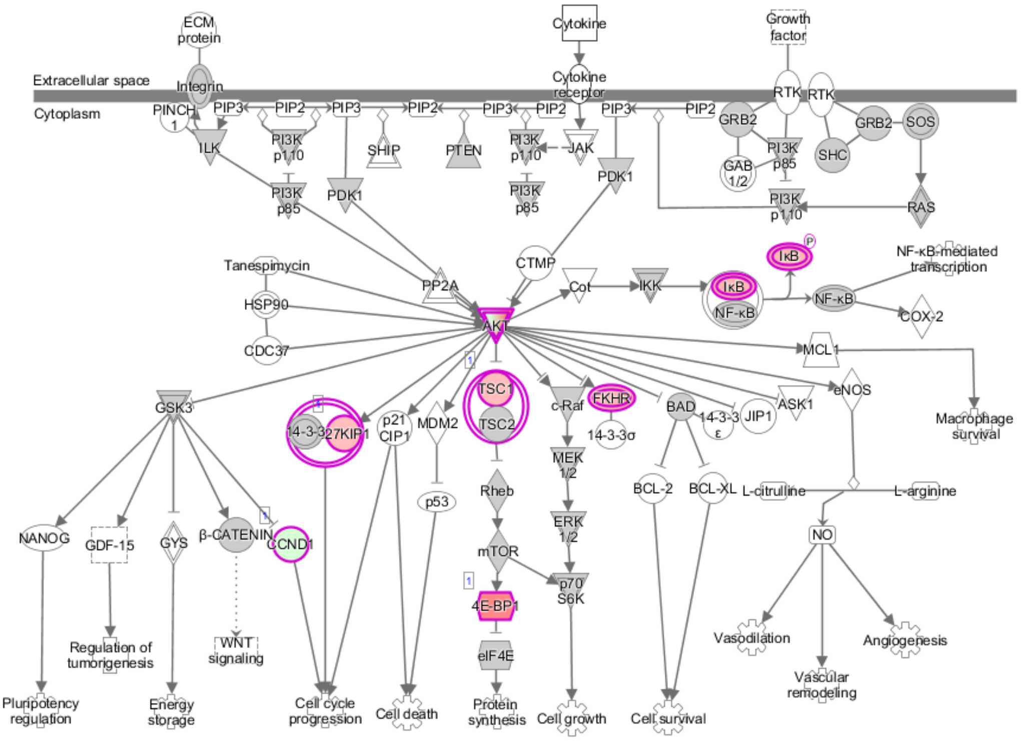

Figure S1. PI3k-Akt pathway of MCF-7 cells treated with the nanocomplex.

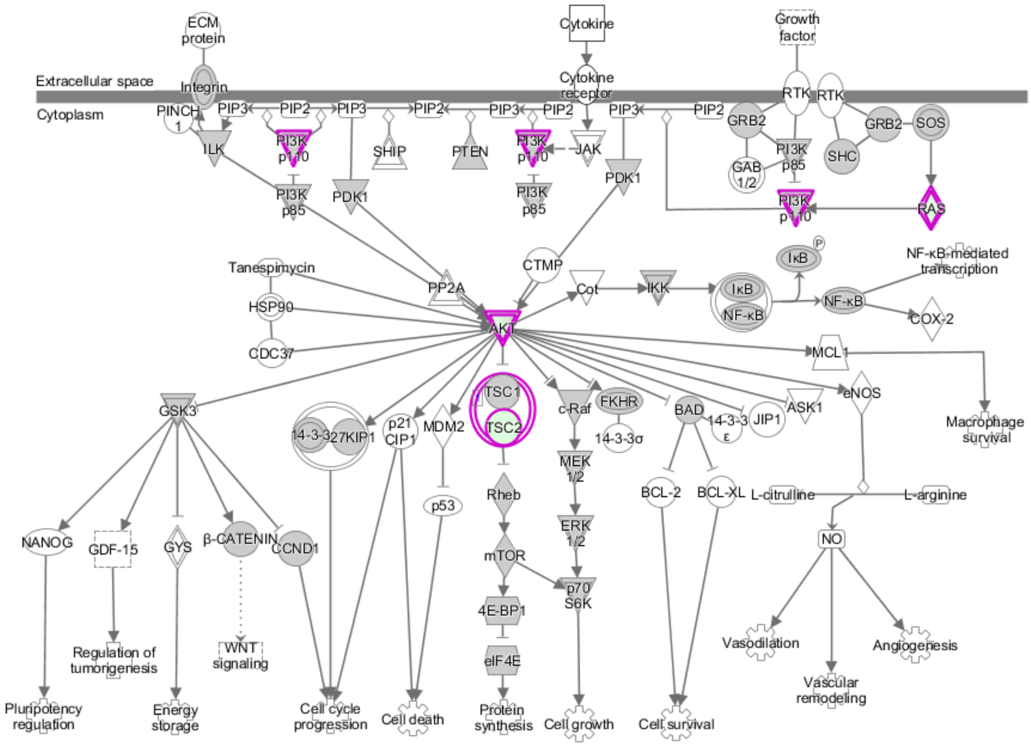

Figure S2. PI3k-Akt pathway of MCF-7 cells treated with the free drug only.

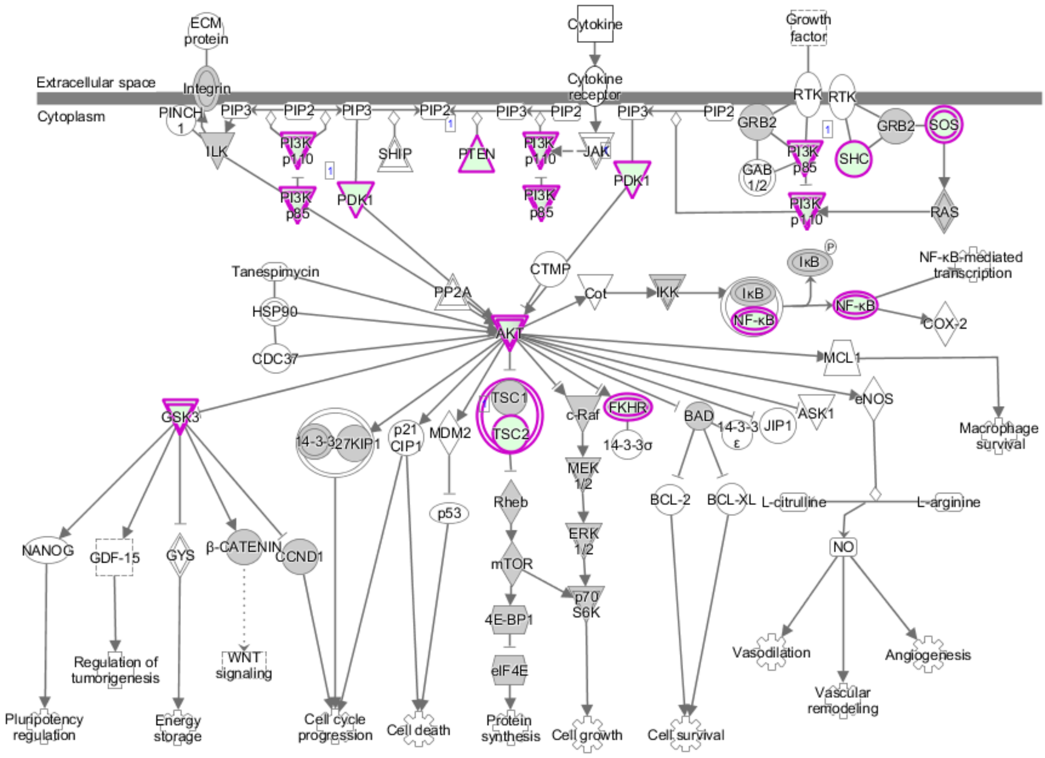

Figure S3. PI3k-Akt pathway of MCF-7 cells treated with GNR only.
